# The COVID-19 System Shock Framework: Capturing Health System Innovation During the COVID-19 Pandemic

**DOI:** 10.34172/ijhpm.2021.130

**Published:** 2021-09-08

**Authors:** Michael Hodgins, Dee van Leeuwen, Jeffrey Braithwaite, Johanna Hanefeld, Ingrid Wolfe, Christine Lau, Emma Dickins, Joeanne McSweeney, Mary McCaskill, Raghu Lingam

**Affiliations:** ^1^University of New South Wales, Sydney, NSW, Australia.; ^2^Sydney Children’s Hospitals Network, Sydney, NSW, Australia.; ^3^Australian Institute of Health Innovation, Macquarie University, Sydney, NSW, Australia.; ^4^Department of Global Health and Development, Faculty of Public Health and Policy, London School of Hygiene and Tropical Medicine, London, UK.; ^5^Institute for Women and Children’s Health, King’s College London, London, UK.; ^6^Integrated Care Project, Sydney Children’s Hospitals Network, Sydney, NSW, Australia.

**Keywords:** COVID-19, Health System Shock, Health Management, Ethnography, Health System Change

## Abstract

**Background:** Coronavirus disease 2019 (COVID-19) has resulted in over 2 million deaths globally. The experience in Australia presents an opportunity to study contrasting responses to the COVID-19 health system shock. We adapted the Hanefeld et al framework for health systems shocks to create the COVID-19 System Shock Framework (CSSF). This framework enabled us to assess innovations and changes created through COVID-19 at the Sydney Children’s Hospitals Network (SCHN), the largest provider of children’s health services in the Southern hemisphere.

**Methods:** We used ethnographic methods, guided by the CSSF, to map innovations and initiatives implemented across SCHN during the pandemic. An embedded field researcher shadowed members of the emergency operations centre (EOC) for nine months. We also reviewed clinic and policy documents pertinent to SCHN’s response to COVID-19 and conducted interviews and focus groups with stakeholders, including clinical directors, project managers, frontline clinicians, and other personnel involved in implementing innovations across SCHN.

**Results:** The CSSF captured SCHN’s complex response to the pandemic. Responses included a COVID-19 assessment clinic, inpatient and infectious disease management services, redeploying and managing a workforce working from home, cohesive communication initiatives, and remote delivery of care, all enabled by a dedicated COVID-19 fund. The health system values that shaped SCHN’s response to the pandemic included principles of equity of healthcare delivery, holistic and integrated models of care, and supporting workforce wellbeing. SCHN’s resilience was enabled by innovation fostered through a non-hierarchical governance structure and responsiveness to emerging challenges balanced with a singular vision.

**Conclusion:** Using the CSSF, we found that SCHN’s ability to innovate was key to ensuring its resilience during the pandemic.

## Background

**Key Messages**

** Implications for policy makers**

- Coronavirus disease 2019 (COVID-19) presents an opportunity to explore the transformation of health systems and determine how different levels of the system coalesce to create and sustain change.
- The COVID-19 System Shock Framework (CSSF) model, based on Hanefeld et al, is an effective tool to map the complex, heterogenous changes that occurred in response to the COVID-19 pandemic.
- Decision-making latitude and ownership of health system processes to foster innovation are vital to supporting systematic resilience in health services.
- By identifying the core values underpinning behaviour and the governance principles stewarding change, we can determine how one health system’s response compares to others.

** Implications for the public**

Australia’s swift response to the coronavirus disease 2019 (COVID-19) pandemic has been a relative success due to its rapid public health and policy response and a relatively low number of cases. This success enabled embedded research to be conducted without impeding the health system infrastructure and personnel during the crisis. Ultimately, our analysis identified many elements that enabled Australia’s health system to respond to the pandemic, including the restructuring of health systems to manage COVID-19, the rapid uptake of telehealth, and the ability to dedicate funding specifically to the response. These changes were underpinned by the principles of equity of healthcare delivery, holistic and integrated models of care, and supporting the wellbeing of the workforce.

Australia’s swift response to the coronavirus disease 2019 (COVID-19) pandemic has flattened the curve of the number of cases reported and resulted in over 15 million COVID-19 tests undertaken.^1^ Australia’s response to COVID-19 was unlike many other countries due to the rapid public health and policy response and the relatively low number of cases. These factors enabled relative success in managing COVID-19, reflected in a low death rate per million people, high ratios of tests per confirmed cases, and a stringent government response during August 2020 when cases in Melbourne were dramatically increasing.^2^ In New South Wales (NSW), Australia’s most populous state, the public health response was highly effective in controlling the early phase of the COVID-19 pandemic.^3^ The resulting low case load has presented opportunities for research *in situ* to determine the factors that support pandemic preparedness and contribute to resilient health systems. This research aims to understand what works well, what should be sustained, and the potential unintended effects of pandemic-prompted system change.

 Older cohorts are more substantially affected by COVID-19; however, it is important the pandemic’s indirect effects on children are not overlooked. Therefore, research should not focus entirely on adult and elderly health systems. The global response to COVID-19 has seen health systems streamlined, communication channels unblocked, models of care modified, and a widespread integrated response to this profound threat.^4^ ‘Mass migration’ of services to telehealth are one of a suite of forced adaptations health services have been required to make. Such adaptations are, in many cases, transforming health service usage and the work practices of thousands of providers across multiple specialties.^5^ These transformations have needed to balance the needs of patients and clients with the physical and emotional well-being of the workforce, with the pandemic placing great strain on healthcare workers.^6^

 It is vital that we understand and share what we have learnt from a health system response to the COVID-19 pandemic. This response can ensure that our health systems not only absorb the impact of future shocks and becomes more resilient after the pandemic, but also to provide a framework to evaluate health system responses in other contexts and countries. The Australian context has enabled embedded research to be carried out without impeding health system infrastructure and personnel. Ultimately, recognising the impact of multiple levels of organisation as part of a systems approach is required for a full understanding of the interventions and policies that might be most effective at strengthening health systems in this challenging climate.^7^

###  A Framework for Learning From the Pandemic 

**Figure 1:**
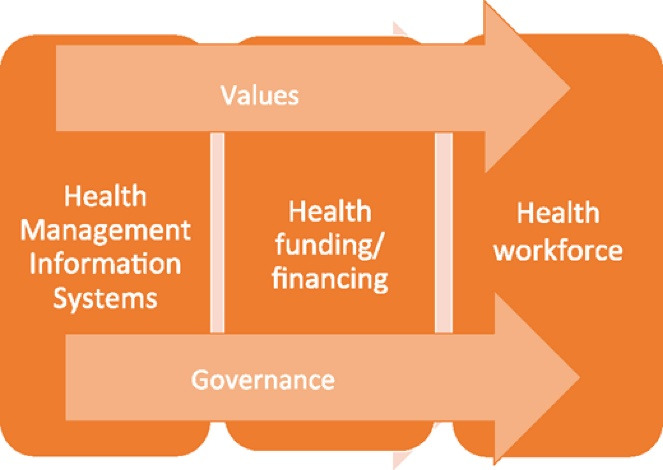
Original Hanefeld et al Framework.^13^

 Our study aimed to systematically collect, collate, and analyse evidence on the direct and indirect impact of COVID-19 on health system functioning from a child health perspective. We also aimed to analyse health system innovations, successes, and challenges in the context of Australian health policy and local needs. We drew on resilient healthcare cases, theories and concepts^8-12^ but focused on systems shock as a conceptual paradigm. To understand, map and explore how the health system has changed, we adapted Hanefeld and colleagues’ model of how health systems respond to shocks (Figure 1), based on the WHO health system building blocks, to create a comprehensive resilience framework.^13^ Hanefeld and colleagues’ ‘learning from shocks’ framework was developed to advance the discussion on how health systems respond to shocks and therefore become more resilient by presenting a framework to study resilience (Figure 1). Their original article analysed four recent shocks in low- and middle-income countries, identifying the dimensions of relevance to health systems’ ability to adapt and respond. They based their framework on findings on system resilience originating in engineering, environmental science, and ecology, and drew on the definition of ‘health systems resilience’ from Blanchet et al (ie, a resilient system can adapt its functioning, absorb shock, and transform to recover from disasters). They based their framework on findings on system resilience originating in engineering, environmental science, and ecology, and drew on the definition of ‘health systems resilience’ from Blanchet et al (ie, a resilient system can adapt its functioning, absorb shock, and transform to recover from disasters).^14-17^ Hanefeld and colleagues’ findings suggest actions in key dimensions determine the extent to which a health system’s response to shock is successful.

 Hanefeld and colleagues’ original article proposed ‘3 plus 2’ critical dimensions. Firstly, they proposed three core dimensions of health system functioning: *health information systems* (ie, having the information and the knowledge to decide on what needs to be done), *funding/financing mechanisms* (ie, investing or mobilising resources to fund a response) and *health workforce* (ie, who should plan and implement it and how). These dimensions intersect with two cross-cutting aspects: *governance* as a fundamental function affecting all other system dimensions and predominant *values* shaping the response and how it is experienced by individuals and communities. Hanefeld et al noted that their framework reflects learnings from specific cases they examined, ‘and it is not intended to be rigid; as evidence increases, new dimensions may need to be added.’

**Figure 2:**
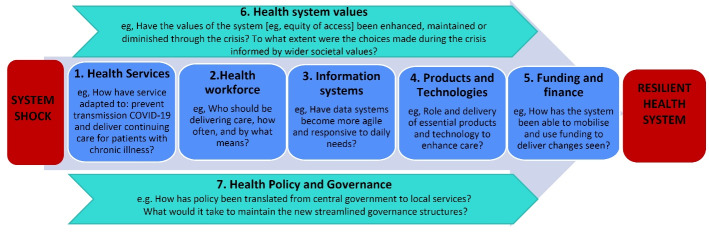
The COVID-19 System Shock Framework. Abbreviation: COVID-19, coronavirus disease 2019.

 Our early reflection on the pandemic response identified several dimensions that prompted us to adapt Hanefeld and colleagues’ model. Firstly, we noted the importance of *health services* and capturing changes to service delivery. We considered this dimension as separate to *information systems*, which transmit knowledge and data across a heterogenous system. Finally, our evaluation highlighted the importance of innovative *medical products and technologies* that enhance care or support emergency management during system shocks. Our adapted framework (Figure 2), the COVID-19 System Shock Framework (CSSF), guided our analyses of how a health system responded to the COVID-19 pandemic.

## Methods

 The CSSF, was applied to study the COVID-19 prompted innovations and changes at the Sydney Children’s Hospitals Network (SCHN). SCHN is the largest provider of children’s health services in the Southern hemisphere. The Network is made up of seven specialised children’s health services: two children’s hospitals; one children’s hospice; a paediatric research service; a newborn & paediatric emergency transport service, a pregnancy and newborn service, and a children’s court clinic. Over 2018-2019 there were approximately 98 000 presentations to SCHN emergency departments, one million occasions of service in a non-admitted patient setting, and more than 157 000 children and young people cared for within the Network.^18^ Although children are not the most vulnerable population to the effects of COVID-19, SCHN needed to establish important infectious disease management systems; adapt their care swiftly to remote models of service delivery; and support peripheral primary paediatric services.

 For SCHN, the shock created by the pandemic over the period from February 2020 to February 2021 specifically involved 462 COVID-19 suspected emergency department attendances by children aged 0-17 years old and 17 inpatient admissions of children with suspected COVID-19 infection. 5422 SCHN staff were tested for COVID-19, with two confirmed cases. A more detailed analysis of the change in inpatient admissions and emergency department attendances from the pre-COVID-19 period (2016-2019) to the COVID-affected period (January 2020-February 2021), is forthcoming in a future publication.

 In response to the COVID-19 pandemic, clinicians, and embedded clinical researchers with the support of a previously established working group, the Medical Workforce Advisory Committee, identified the need to capture the rapid health system innovation of SCHN to ensure that COVID-19 was a transformative shock. The CSSF framework provided the deductive schema for data collection. Using this framework, we mapped the innovations and initiatives that were implemented across SCHN during the pandemic using qualitative ethnographic methods. This involved a member of the research team becoming an embedded researcher within SCHN, shadowing members of the emergency operations centre (EOC) over a nine-month period. This embedded researcher model has been used successfully in previous research as a response to waning enthusiasm towards academic research and intrusive methodologies and the importance of acknowledging local complexity.^19,20^

 Due to the nature of the pandemic and the need to limit contact, the majority of the EOC operations occurred virtually. As such, shadowing involved a member of the research team phoning in to EOC teleconferences and compiling observational fieldnotes documenting the decision-making process as it happened. During the initial stages of the pandemic these were held twice daily. By June/July of 2020 these were reduced to twice weekly and by September the regular EOC teleconference briefings were ended due to the stability of the COVID-19 pandemic in Australia. Overall, 48 meetings were attended by a member of the research team. To complement the data collected while shadowing, our team also reviewed clinic and policy documents pertinent to SCHN’s response to the COVID-19 pandemic including network wide communications to staff, minutes from EOC meetings where the response to the COVID-19 pandemic was discussed, and policies created during the COVID-19 pandemic. These documents were reviewed to supplement the analysis of other qualitative data, for example, by providing clear timelines for staff-wide communication and changes in policy.

 To augment these data, we also conducted 42 interviews and focus groups with key stakeholders, including clinical directors, project managers, frontline clinicians, and other personnel involved in rapidly implemented innovations across the Network. Recruitment of participants followed a snowball sampling process, with interview participants identifying other participants for interviews; a method used to good effect in previous work.^21,22^ Semi-structured interviews and focus groups explored how clinical and organisational practices changed because of the COVID-19 pandemic and the positive and negative consequences of the changes to clinical and organisational practices. Interviews and focus groups followed a semi-structured interview guide with broad questions. The prompts we used during interviews and focus groups were guided by the CSSF, specifically considering changes to:

- Health services
- Health workforce
- Information and communication systems
- Medical products and technologies
- Funding and finance
- Health system values
- Health policy and governance

Additionally, we drew from Braithwaite’s notion of hidden resilience and brittleness in healthcare to inform interview and focus group prompts, considering the relationships between the spatial, social, and material arrangements that make up innovations.^23^ We used the principles of qualitative data saturation to determine the sample size of interviews and focus groups by way of a process of preliminary coding and analysis to determine new and repeating themes and innovations.^24^ Interview recordings were purposefully transcribed and coded deductively using the CSSF and inductively, following Braun and Clarke’s approach to thematic analysis.^25^ Our analysis was aided through close collaboration with SCHN staff, with members of the SCHN EOC and other clinical staff verifying and critiquing findings, identifying pertinent themes and innovations not readily captured during data collection.

## Results

 Our results identified innovations implemented within the SCHN, coding them according to the CSSF (Table).

**Table:** Innovations Mapped to the COVID-19 System Shock Framework

**1. Health Services**	**2. Health Workforce**	**3. Information Systems **	**4. Medical Products and Technology **	**5. Funding and Finance**	**6. Health System Values **	**7. Health Policy and Governance**
Assessment clinic	Working from home support, including equipment and virtual private networks	Daily email update	Telehealth and virtual care	The COVID-19 fund	Enabling equity of access through remote delivery of care	Rapidly formed EOC
COVID-19 Positive Outpatient Response Team	Infectious disease training for staff, including cleaning staff	Staff intranet information page	Remote monitoring systems	Collaborative care models splitting private and public funding	Acknowledging and supporting the emotional wellbeing of staff	Adaptive structure allowing working groups to confront emerging challenges
COVID-19 wards	Training and education for potential staff redeployment (eg, training paediatric clinicians for adult ICUs)	Information web page for families	E-gate automatic concierge screening	Initiating government funding of telehealth	Continuing care in the face of uncertainty	Transparent and consistent communication
SOPs providing an integrated repository of standard care practices	Delivering education remotely	PPE Dashboard	QR codes for hospital entry		Continuing preparedness for infection rate surges	Breaking down silos across the Network through transdisciplinary networks
Medical students included as part of the pandemic workforce supported by adapted workforce ie, extending visas	PPE training modules for donning and doffing PPE	Published emergent findings from global research papers and international collegial connections	Home spirometry devices		Holistic values within transformed care practices	
		Emergency response matrix			Equity pathways	
		Patient flow dashboard				

Abbreviations: COVID-19, Coronavirus disease 2019; PPE, Personal Protective Equipment; ICUs, intensive care units; EOC, emergency operations centre; SOPs, standard operating procedures.

**Figure 3:**
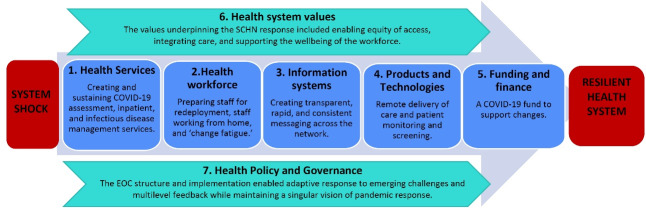
Principles Guiding System Changes Mapped to the COVID-19 System Shock Framework. Abbreviations: COVID-19, coronavirus disease 2019; SCHN, Sydney Children’s Hospitals Network.

 Further, we considered thematically the principles guiding change within each dimension of the CSSF and the principles cutting across all elements of the framework, provide as a thematic map in Figure 3.

 The two cross-cutting elements of the framework, values, and governance, are also addressed as they relate to the response. The underlying values, encompassing political, societal, personal, and professional ‘moral landscapes’ on which difficult decisions are negotiated, cutting across a response to shock are typically overlooked in structural and input analyses of crises response. In our analysis these values critically shaped SCHN’s response to the pandemic, with principles of equity of healthcare delivery, holistic and integrated models of care, and care for the workforce underpinning all system changes. These values were driven primarily from the governance structure, with the established EOC affecting the operation of all system dimensions.

###  Health Services 

 The nature of COVID-19 has necessitated rapid reshaping of health services, as the system has been forced to develop new ways of operating to accommodate large scale testing and social distancing.^26-28^ The rapid initial infection rate in NSW prompted the SCHN Executive to establish the EOC in March 2020. The EOC was tasked with overseeing the management of SCHN’s response to the pandemic, overseeing the setup of COVID-19 treatment areas, wards, and assessment clinics at both hospital sites, and reconfiguring the workflows of emergency departments to triage and manage potential COVID-19 patients. The COVID-19 ward management teams developed standard operating procedures (SOPs) that directed practice in response to NSW Health and organisational directives. Over 100 SOPs were developed from March 2020 on situations ranging from deteriorating patients within the COVID-19 ward, concierge screening practices, and infectious disease management. The SOPs were designed to standardise COVID-19 ward practice according to existing evidence-based literature. To assist this process key departments across the Network provided resources in relation to treating common presentations. A General Medicine simulation team was developed to run weekly interdisciplinary COVID-19 simulations involving deteriorating patients that ended in a cardiac arrest and code blue call. Using an innovative Pause-and-Discuss simulation approach in-situ on the COVID-19 ward, the simulations helped to disseminate critical information embedded in the SOPs and provided opportunities for real-world clinical practice in a safe learning environment. This improved the confidence and preparedness of staff, building their capacity to adapt to rapid change.

*“(The simulations) were a huge success, they were on the ward so we actually could… run a simulation like it was going to happen… We picked up important things, running a resus(citation) for a COVID patient was going to be hugely different than what we had ever done before*” (COVID-19 ward staff).

 Patients and carers on the COVID-19 wards were required to adhere to strict isolation guidelines leading to increased frustration and emotional distress for carers who felt less supported in the isolation wards. The SCHN social work department implemented a wellbeing check in via telephone into the isolation rooms to address this challenge, which was reported to improve patient and carer wellbeing according to ward nursing unit managers and medical officers.

 COVID-19 assessment clinics operated at both hospital sites for children accompanied by a parent or guardian. Additionally, SCHN established a COVID Positive Outpatient Response Team. This team, made up of senior staff from within the Ambulatory Care unit, worked closely with the Network’s Infectious Diseases teams and Public Health units to provide virtual care to COVID-19 positive children in their homes. This team also integrated with social workers and mental health clinicians to provide physical and psychosocial care for young children who tested positive for COVID-19 and their families. This model was one of the first paediatric COVID outreach services provided in NSW. Teams within the Network also recognised the importance of maintaining initiatives beyond the scope of typical healthcare. For example, the Chronic Illness Peer Support program launched ChIPS 2.0 to maintain an online engagement with chronically ill patients in the form of video chats, webinars, and online gaming. Additionally, the Network’s palliative care team hosted weekly virtual play sessions for children engaged in palliative care services and their families. These initiatives exemplified holistic approaches to healthcare, and teams identified these initiatives as important to maintain.

###  Health Workforce

 The health workforce is both essential to pandemic response and the most vulnerable.^29,30^ Global research has pointed to the need to test frontline health staff as a priority, provide ample personal protective equipment (PPE), reinforce the importance of social distancing, and provide appropriate training, knowledge and protocols to follow.^31^ To maintain health services during the pandemic SCHN redeployed staff and planned for surges, for instance redeploying physiotherapists into intensive care units (ICUs); theatre nurses swab training; supporting adult ICU training and preparing research staff members with clinical experience for redeployment into clinical roles. In addition to the management of potential surges, the pandemic necessitated changes in work practices. Where possible SCHN staff worked from home and in person meetings were capped or moved online, supported by the IT teams and technology. Common areas within both hospitals erected social distancing messaging and reduced seating. SCHN took the initiative to identify staff vulnerable to COVID-19 infection (including staff who were over 65 and aboriginal workers) and these staff were invited to be redeployed or work from home where possible. Special COVID-19 leave (paid) was provided for staff with symptoms requiring a COVID-19 test or with the illness. Given the limitations placed on students and junior staff attending the hospital on site, personnel within the Network were required to innovate with how they delivered continuing professional development, for instance using telehealth meetings, simulation, online education modules, and podcasts. Some participants noticed that education for more junior personnel could be improved in future:

*“We need to spend some time thinking and collaborating with the clinicals schools and universities to work how we can do this differently … We already are talking about simulation we want to include in education, which is an under resourced area of our organisation*”(staff specialist).

 While empathy and positivity, potentially due to the low COVID-19 numbers, buoyed SCHN’s response to the pandemic during the preliminary months of the pandemic, interview participants have noted that change fatigue has resulted in weariness among staff. Additionally, teams with personnel working regularly from home noted the long-term effects of isolation, as one nurse unit manager attested:

*“People that are working from home, now I feel the need to check in on them because they don’t necessarily feel like they’re part of the team, I think they feel isolated*” (department head).

 Additionally, some clinicians noted that virtual care was an add-on to existing clinical work, as opposed to supplementing existing face-to-face work, extending the energy and time required for practice:

*“With telehealth … I’m finding that I’m more exhausted… Telehealth and virtual platforms have enabled us to continue our work as usual, but it’s also led to us actually taking on more …. (Now) we have back-to-back meetings” *(staff specialist).

 NSW’s limited case numbers have meant that many staff have been able to return to work on site, however managing the longer-term impacts of COVID-19 workforce innovations on workforce will be an important consideration going forward. Many participants have since identified the importance of maintaining workplace flexibility where possible and identified supports, such as technical and hardware for staff to continue to work effectively from home, which is reflected in continuing the Medical Workforce Advisory Committee evaluation.

###  Information Systems

 A vital element of SCHN’s response to the COVID-19 pandemic was a consolidated effort to coordinate and disseminate important information, which has been shown to be vital for emergency preparedness despite limitations in many health information management systems.^13,32^ The need to inform staff across the Network proved a necessary step to combat the disparate and, at times, panic-inducing rate of information being disseminated rapidly across news outlets, social media, and government announcements in relation to the pandemic:

*“There was just such a rapid fire of information when COVID hit in March, particularly regarding the stuff that was happening in Italy and elsewhere. Everyone had questions and, certainly at the beginning, the executive team and managers didn’t always have easy answers*”(COVID-19 hospital site lead).

 Consolidating the, at times, frenetically paced influx of new information about the pandemic represented a significant challenge for the Network given the disparate facilities. This challenge was met by key actions undertaken by the EOC and executive team to support the dissemination of consistent information.

 The daily COVID-19 update was posed by communications and executive team members to manage the rapid influx of information surrounding COVID-19. The daily COVID-19 update provided a consistent line of communication from the executive and emergency operations teams to the multitude of staff at the Network. The COVID-19 daily update proved an acceptable and effective method of delivering important COVID-19 information according to an audit of staff acceptability of the communication method. In addition to the dissemination of important information, SCHN developed key monitoring tools including a PPE dashboard that indicated the levels of PPE stores available, a patient flow dashboard to track individuals who tested positive to COVID-19 journey through the hospital, a literature repository and summary that added COVID-19 clinical literature consistently and an emergency response matrix. The combination of these communication initiatives proved a vital aspect of the emergency management, giving staff confidence and assurance to do their job effectively:

*“There was a lot of transparency and consistency in terms of information coming from the top, and with the daily update and the growing body of information for staff and families it just meant people had one source of truth for the questions they had*” (nurse unit manager).

 Much of the pandemic-related innovation was predicated on the responsive and coordinated communication strategy developed by SCHN.

###  Medical Products and Technologies

 Remote delivery of care and patient monitoring and screening underpinned COVID-19 prompted technological innovation. SCHN were required to switch to telehealth and virtual care for clinical departments where viable, a transition that was well coordinated, particularly through collaboration between multidisciplinary and external departments. Through this collaboration, expertise could be drawn from respective departments to ensure accurate and timely telehealth information was disseminated across the Network. Telehealth COVID-19 Implementation Guides, appointment letters, billing flowcharts, and help sheets/troubleshooting guides were developed and made available electronically; virtual meeting rooms were created for departments to conduct their telehealth consultations; and equipment was procured including webcams, headsets and speakerphones expediting uptake. Remote care included, for example, home spirometry. Spiro Home devices are a portable Smart spirometer compatible with Smartphones to enable clinicians to remotely determine if a patient’s lung function has declined or if their current treatment therapy is working as expected. Managers used COVID-19 as an impetus to rapidly transition to telehealth, acknowledging the difficulty of this task outside of ‘pandemic conditions:’

*“The pandemic has been an impetus for us to implement changes in our team that we’ve wanted to do for a long time, delivery of care through telehealth was something we wanted to do for a while now” *(department head).

 The implementation of virtual care across the Network was further enabled by the Virtual Hospital program of work, an initiative that began pre-COVID-19 in January 2020 with COVID-19 bringing forward its implementation.

 In addition to the implementation of telehealth, patient flow through the hospitals changed dramatically. SCHN was one of the first NSW services to set up a QR code to enable people entering the two main hospital sites to be screened on entry and check in. The pandemic dashboard was designed to receive COVID-19 tests and monitor positive cases through the hospital to enable workforce and clinical planning to direct tests, services, and management. This dashboard has utility beyond COVID-19 to monitor other communicable diseases through the hospital, either in real-time or retrospective analysis of hospital response. SCHN partnered with a local University to develop an automated concierge process involving an electronic gate (e-gate) and thermal camera to improve the flow of screening newly arriving visitors to the hospital and reduce concierge staffing.

###  Funding and Finance

 Adequate and well allocated funding has the potential to shore up health systems in the face of shocks and conversely exacerbates the negative impacts of shocks when unpredictable or limited.^33^ To support the response to COVID-19 the NSW state government established a COVID fund for health services. COVID-19 funding priorities within the Network included assessment clinics, PPE, cleaning, concierge, and the Clinical Communications program, Medtasker. The allocation of COVID-19 funding had a small degree of leniency, with any cost exceeding $10 000 requiring approval from the Ministry of Health. This funding enabled many of the innovations that occurred within the Network and proved a workaround for gaps in funding for necessary services. Additional sources of funding to support innovation included research funding, for instance, a research grant providing funds to support the construction of the e-gate. In March 2020, new COVID-19 telehealth Medicare Benefits Schedule items were introduced that enabled telephone and videoconference consultations to be bulk-billed for patients residing in both metropolitan and rural locations. This allowed equitable access of care for patients and ensured vulnerable staff could continue to work and continue to provide care without be placed at risk.

###  Values

 Values crystallise in the face of health emergency, dictating policy-making and the way services are delivered.^34,35^ COVID-19 has brought to the fore many values in the health service landscape and society in general, admirably those such as personal responsibility and empathy have enhanced the way the pandemic has been managed on many fronts.^36,37^ Through our analysis we identified three core values as underpinning much of the response to the pandemic including maintaining or enhancing equity of access to healthcare, breaking down disciplinary silos to integrate care, and supporting workforce wellbeing.

####  Enabling Equitable Access of Healthcare

 When asked about how system change was enabled so rapidly and effectively, many participants spoke of the inherent qualities of the workforce to continue to deliver care for children and families in need. Participants spoke of the necessity to keep services up and running and to reach patients and families most in need of healthcare:

*“It’s just what we do. We are wired to make it work no matter what challenges we face”* (registered nurse).

 Participants cited telehealth as one innovation capable of enabling equitable care. Many of the participants stated the importance of a hybrid model of care supported by telehealth in a post-COVID-19 landscape to support equity:

*“Delivering services remotely is great, but it doesn’t work for everyone, there are some patients that it just doesn’t work for, for whatever reason … Moving forward I think we will need to combine telehealth and seeing patients in person” *(department head).

 Despite an imperative to enable access, many participants commented on the challenges around policies limiting physical access to the Network sites.

*“A lot of families have found that difficult. They understand the reasoning and are cautious themselves about people bringing COVID into the hospitals, but that was a decision that was met with some anger and frustration*” (department head).

 Participants noted that the limitations on parents, carers and siblings attending the hospital with their children were met with some frustration, especially for parents and carers of children with a life-limiting illness. These comments underscored demonstrations of participants valuing enhanced access to care.

####  Breaking Down Silos and Integrating Care

 Early lessons from the first wave of COVID-19 which affected countries such as Singapore, South Korea, and Japan were that ‘integration of services in the health system and across other sectors amplifies the ability to absorb and adapt to shock.’^38^ The experience at SCHN mirrors this finding, with integration of the health service response across the Network proving an important element of emergency management. The response of SCHN demonstrates that transdisciplinary communication and coordination, for instance with cross network and interdepartmental representation in governance and the development of many cross-disciplinary SOPs, is vital to emergency management in health systems. The ‘breaking down of silos’ and empathy for each other shared across the Network was a key theme identified as part of the analysis of the data:

*“Initially it was a really scary time, looking back on it… breaking down those silos and being able to collaborate, giving nurses the confidence to nurse things that they would never think they would be able to nurse, has been a huge achievement” *(COVID-19 ward nurse unit manager).

 Much of the innovation within the Network demonstrated an ability to transcend friction and the typical barriers of interdisciplinary collaboration. Personal and professional moral imperatives of ‘coming together’ and ‘breaking down silos’ in the face of fear and anxiety pervaded the data within a vast and disparate Network to support each other and achieve change.

*“What enabled the rapid change is that everyone had a shared feeling of ‘This is going to affect us all equally. We need to all chip in.’ There was, and still is, a great deal of empathy across everyone in the organisation ”* (EOC member).

 Participants also acknowledged their role as global citizens in response to a pandemic affecting the wider world:

*“We were just seeing what was going on around the world, especially in Italy and we thought this is affecting everybody, it’s going to be us soon” *(administrator).

 Facilitating empathy through acknowledgement of the wider healthcare community as a collective body and demonstratable representation of disparate disciplines within executive decision making is an important consideration for rapid change and innovation.

####  Acknowledging and Supporting the Wellbeing of the Workforce

 A social phenomenon that emerged from the initial response to the pandemic from a health workforce perspective was immense external support prompting ‘resilience amid challenges.’ To support the wellbeing of the workforce, SCHN provided internal organisational psychology support for teams, engaged in ‘wellbeing checks’ and team check-ins and currently provides an onsite psychologist available across the Network. Many staff acknowledged the necessity of their work, and the potential difficulties working through a pandemic:

*“When you work in healthcare, you have to be prepared to put yourself at greater risk than most people working would put themselves at”* (registered nurse).

 From the initial stages of the pandemic, health-care providers identified many sources of social support and used self-management strategies to cope with the difficult situations COVID-19 presented.^39^ Australia generally witnessed an outpouring of support for healthcare workers, who were viewed as selflessly battling on the front lines of the pandemic.

 One example of the outward displays of support during COVID-19 was a kindness tree, an initiative developed by an Adolescent Unit in one of the sites. The kindness tree was a large mural providing opportunity for patients, visitors, and staff to contribute messages on leaves instilling positivity and acknowledgement of all staff, patients, and families on the ward.

*“There is great team spirit here at the moment. Everyone seems to have a really positive can-do attitude” *(department head).

 Overall, the understanding among the health community, particularly from the international community, that the pandemic required a strong and resilient health system response, coupled with the general success of the local response meant that the SCHN community were able to respond positively to the pandemic. However, change fatigue, lapses in attention and a lack of necessity to use the pandemic preparedness process due to the low infection rate locally in the last few months mean that SCHN will need to continue to provide sustained COVID-19 support for their community.

###  Governance 

 The governance of health systems has attracted recent attention based on the argument that good governance leads to improved health outcomes.^40,41^ Abimbola and colleagues have argued for multi-level or polycentric governance where in which failure at one level can be assuaged at other levels of governance, including by non-government health system actors. The governance of SCHN provides good support for this argument.^42^ The EOC structure and implementation enabled adaptive response to emerging challenges and multilevel feedback while maintaining a singular vision of pandemic response. The EOC was formed from experienced senior administrative and project management staff, representing a cross-section of the organisation, chaired by the Network Incident Controller (MM), and attended by the SCHN Disaster Controller. A key principle of the EOC that fostered collaboration was the openness of the organising structure, rejecting hierarchical linearity in favour of an ‘open team’ where members interacted to spread and generate collective knowledge across clinical and organisational domains. Personnel were assigned positions in addition to their fulltime roles, or in place of substantive roles that had paused due to the pandemic. Many of these staff had never met each other or worked together and there was a steep learning curve for support staff not previously exposed to Emergency Management. Despite this, the EOC were able to collaborate effectively and respectfully to lead the SCHN response to the pandemic. As one EOC member suggested:

*“All members were active contributors, all perspectives welcomed, issues able to be raised by all members without hierarchy or ‘politics.’ There was a strong sense of striving for common goals of keeping patients, families and staff safe, while making the necessary adjustments to deliver best possible care*”(EOC member).

 Working groups were formed to address more specific challenges, including those tasked with managing PPE, the COVID-19 assessment clinics, research and quality improvement, and surgery and anaesthetics.

 The leadership of the EOC was vital to the management of, at times, chaotic information flow throughout the Network. An important part of the emergency governance structure was lines of communication traveling both from external government and other health advisory bodies and executive members to staff, and staff on the ground being able to communicate back to EOC to inform their decision making: “*EOC’s role was massive … and there was a (communication) conduit both ways.*” In some instances, SCHNs actions and responses to external agencies helped to inform state-wide strategies and responses to the pandemic, evidencing the non-siloed, collegial approach of the NSW and Australian health system. COVID-19 site leads at each of the main hospitals sat within the EOC and could feedback what they were seeing and hearing into the decision-making mechanisms.

 The team in charge of SCHN pandemic response have recognised the need for ‘being open and honest,’ and garnering insight from frontline workers as core components of the pandemic response to allay fears and build trust. The systems of information management have been praised by many of the participants of our study, particularly for its ability to rapidly deliver important information:

*“We were responding, in many ways, more quickly than the Ministry (of Health), in the sense of, we’re on the ground, we know immediately what we need on the ground*” (communications manager).

 In staff feedback surveys since the commencement of the pandemic, staff have praised the value of the information disseminated early in the pandemic. Early education, for example, in PPE training ‘prepared them to respond to the pandemic.’

 Some participants criticised aspects of governance and leadership, specifically for instances of poor communication and not providing explicit guidance during particularly stressful periods of the pandemic. One participant, for instance, noted communication for clinicians was not always clear:

 “*At times we were not well communicated with, despite gradually becoming a very clear process around information being disseminated … Your hierarchy of line management means some of that information is diluted to clinicians of all levels … and that can be complicating … We felt often confused about what was expected*” (staff specialist).

 This clinician noted that some roles had better informal links to executive team members, which provided greater ‘inside access’ to decision making and information. This same clinician suggested more real-time communication tools, specifically an SMS all user messaging service, would have negated this challenge.

## Discussion

 Our CSSF model allowed a systematic analysis of change at multiple levels of a one of the largest paediatric health services in the southern hemisphere. As part of our analysis, we identified the key innovations SCHN implemented in response to the pandemic. We also explored the values and governance structure underpinning these initiatives. COVID-19 presents an opportunity to explore the transformational change of health systems, to determine how different levels of the system coalesce to create and sustain change. The CSSF model, based on Hanefeld and colleagues’ work,^13^ has proved a useful tool to map the complex, heterogenous changes that have occurred because of the COVID-19 pandemic. Principally, this framework allowed us to consider how SCHN innovated across the different functions of the system to meet the challenges faced during the COVID-19 pandemic. Hospital stress plays a crucial role in addressing pandemics despite national and international authorities underestimate this factor.^43^ Rather than merely coping with the COVID-19 pandemic, SCHN’s ability to innovate enabled the system to demonstrate its resilience and change in the wake of this system shock. The limited COVID-19 case numbers within Australia gave us the opportunity to consider innovations created by clinicians and other staff in the face of system shock *in situ*. Rather than introduce externally-developed, if not superimposed, innovations that were contingent on rigorous evidence many of the changes that have occurred have come from staff on the ground determining immediate need and responding accordingly. It is important to ensure these solutions are in line with emerging evidence and evaluated accordingly as the pandemic response progresses.

 This work provides a contribution to the concept of health system resilience, particularly the response to shocks, which may prove a crucial consideration in global health discussions given the pandemic.^38^ By identifying the core values underpinning behaviour and the governance principles stewarding change, we can determine how a health system response compares to others. Health system resilience is dependent upon actors and networks whose choices and actions.^44^ These actors within the network need to have the agency to influence the health system, as identified in previous scholarship of resilience.^45^ The absence of decision-making latitude and ownership of health system processes can hamper systematic resilience.^46^ Vital to SCHNs response was the ability to provide a voice to many actors with the system, through a non-hierarchical governance structure, a fostering of interdisciplinary collaboration, and, crucially, acknowledging and supporting the wellbeing of the workforce. We agree with Abimbola’s definition of resilience in health systems as going beyond the notion of coping to strengthening, in the case of the pandemic response: strengthening through innovation.^47^

 Our analysis supports preliminary commentary in the wake of the pandemic identifying the importance of flexibility and thoughtful stewardship of resources as key components of pandemic managment.^48^ Although existing research has yet to explore in depth how this is managed from both meso and micro perspectives. Additional elements of the SCHN response to the pandemic that align with resilient health systems as identified by Chamberland-Rowe et al include the constant monitoring of system capacity and performance, the ability to mobilise funding, and multisectoral coordination.^49^ Again, our work explored not only this system level decision making, but also the impact on health staff on the ground. Future research should consider further how differences in the governance structure of response to crises response shape the response. What is absent from this evaluation is direct community engagement as part of SCHN’s response, which may prove a limitation, particularly given the challenges faced by families to attend hospital with children.

 It is important to highlight that the adaptations and innovations captured as part of this research, indeed even those that suggests system resilience, do not implicitly suggest strengthened healthcare and improved health per se. As Topp notes, resilience and improved health and equity can be conflated, with ‘the capacity to adapt and implied resilience it conveys becom[ing] equally or more important than whether that adaptation and resilience produces improved health.’^44^ For instance, delivering care in the face of adversity through positive deviance may on the surface be seen as a form of resilience, but it has been demonstrated that long term reliance on such behaviours is likely to contribute to systemic brittleness, not resilience, since: *‘*one set of personnel, those at the outermost level […] bear a disproportionate burden in supporting the system, leaving them overstretched and in a potentially very unstable situation. If circumstances were to further change or these personnel are no longer able to cope, the entire system could break down very quickly.’^50^

## Conclusion

 The COVID-19 pandemic presented an opportunity to explore the transformation of health systems and determine how different levels of the system coalesce to create and sustain change. The CSSF model, based on Hanefeld et al, is an effective tool to map the complex, heterogenous changes that occurred in response to the COVID-19 pandemic. Decision-making latitude and ownership of health system processes to foster innovation are vital to supporting systematic resilience in health services. By identifying the core values underpinning behaviour and the governance principles stewarding change, we can determine how one health system’s response compares to others.

 The principal limitation of this work is that it was not conducted in a system that was particularly stretched by the pandemic. Taking the CSSF framework up in contexts under more pressure by the pandemic may prove telling in the contribution of this framework. Another limitation of this work is the lack of direct evidence confirming the impact of policy and structural changes on the improvement or maintenance of healthcare quality at SCHN. Our study, though independent, was substantially enhanced by support from key leaders from SCHN regarding capturing these health systems change in response to the pandemic. Another limitation is that perspectives more critical of the governance response to the pandemic were less apparent in interviews; however, this may have been due to a sampling bias of clinicians and staff less praiseworthy of the SCHN response to the pandemic also less agreeable to participating in the study, particularly with the embeddedness of the research within team. However, the research team actively sort differing perspectives by asking participants following interviews if they knew of personnel with alternate views, however no clinicians were identified as part of this process.

## Ethical issues

 Our study received ethics approval from the SCHN Human Research Ethics committee (HREC reference:2020/ETH01432). All interviews were with consenting participants informed of the study aims and details.

## Competing interests

 The SCHN has funded this present manuscript. The authors: DvL, CL, ED, JM, MM, and RL are employed by the SCHN.

## Authors’ contributions

 All named authors have made substantial contributions to the conception or design of the work; or the acquisition, analysis, or interpretation of data for the work; drafting the work or revising it critically for important intellectual content; final approval of the version to be published; and agreement to be accountable for all aspects of the work in ensuring that questions related to the accuracy or integrity of any part of the work are appropriately investigated and resolved.

## Disclaimer

 The views expressed in the submitted article are our own and not an official position of the institution or funder.

## Funding

 This project was funded by the SCHN.
